# Chronic Disease Forces, or Miasms

**Published:** 1892-04

**Authors:** Edward Cranch

**Affiliations:** Erie, Pa.; Bureau of Homœopathic Philosophy, I. H. A.


### CHRONIC DISEASE FORCES, OR MIASMS.

EDWARD CRANCH, M. D., ERIE, PA.

(Bureau of Homœopathic Philosophy, I. H. A.)

The above subject, in substance, was proposed to the writer by the late Chairman of this Bureau, Dr. William A. Hawley, but in view of the able papers on the subject that have appeared during the year, it will be advisable to confine the present essay to the few points that have most engaged the writer's mind.

It is universally conceded that there are chronic and incurable diseases, and it is also well known to Hahnemann's students that there are no disease-entities, as separable existences, but that every disease is an expression of the disturbed dynamis, or universal life-force in this or that individual subject. Even in mechanical disorders, fractures, lacerations, repletions, etc., the disturbing symptoms by which we know the organism is disturbed are of similar dynamic origin.

Now where, in our philosophy, is the bearing of this hypothesis upon the law of Homœopathy, and what ought it to teach us in regard to the chronic, or incurable disturbances of the life-force known as miasms?

We can see that all disease is in the disturbed reception, by the subject of disease, of that constant flow of life that comes every moment from the Creator, just as heat and light come to nature from the sun of the material world.

What kind of disturbing causes or hindrances to the orderly life-current determine whether the disease shall be acute, chronic, or incurable, and how do homœopathic remedies turn aside such disturbances? The orderly life-current comes from healthy brains and ganglia, into healthy foods and solids, causing the whole man to grow and perform his functions, voluntary and involuntary, in an orderly manner—that we call health.

What disturbs it? Let us suppose that the child, or the man or woman, eats too much. The *prima viæ*, the largest tubes in the body, the stomach and intestines, are engorged, in whole or in part. From them the various absorbents and distributing vessels are crowded in turn, and the violence of the disease is in direct proportion to the fineness of the vessels and fibres involved, for even death may be the penalty of an excessive meal, so embarrassing the outflow of the normal fluids that even the brain swoons, and life passes beyond recall, not having room for action. It is a universal truth that influx is proportioned to efflux; if the electric wire has no ground current or its equivalent, it will not flow off; if a man has no activity he dies; if life has no circulation it ceases to animate the body.

Now, such is the elasticity of the bodily economy that it will constantly and continually endeavor to right itself from every disturbance, never ceasing its struggles to attain free conditions, but not disdaining to accept altered conditions, if it cannot find or force the way to perfect conditions.

And right here is the distinction between acute and chronic diseases. Acute diseases soon allow the vital-forces to flow again in their former channels, but if acute conditions frequently return, or if the original disturbing force is of such a nature as to effect distortion and perversion of the vessels and cellular elements of the body, then health cannot be restored except by such relief to the disturbance as will enable the distorted and perverted cells and vessels to use their share of the ever-acting life-force properly.

In other words, the healing power is not in the medicine, or in the cell, or in the body, but solely and entirely in the vital force we call life, acting on the body by means of constant creative energy.

The disease force acts from without, from the grosser vessels to the finer, the vital-force overcomes the disease-force, if at all, from within, insidiously, slowly, and never perfectly, if the outgoing channels have been permanently warped.

How does the homœopathic remedy effect restoration to health?

In the first place, by its homœopathicity it finds the very path of the disturber, and secondly, by its subtle dynamic quality it is able to go to the very citadels of the life-forces, in the ganglionic and cortical glands, and summon them to the fight, which is prompt and easy, or slow and difficult, just in proportion to the acquired nature of the cells and vessels that are to be the scene of the conflict.

By inherited or acquired malformations of structure a disease is rendered chronic, and is called a miasm, when such malformations are latent or diffused, altering the life-force in every function of life, and seriously diverting or thwarting it in its efforts to throw off acute diseases that may occur at any time in addition to the chronic or latent state of the vessels just called a miasm.

Such miasms are like the nail in the heart of the oak, warping the growing fibres, and leaving the door open to early decay.

Remove the miasms, if you can, but remember that the old oak must often be propped, and not too deeply probed lest we hasten its downfall. Youth is the time to fight miasms and all other bad habits. The devil is said to smile when a man of forty talks of reforming. So a miasm, whether from sycosis, psora, vaccination, or, it may be, any microbic infection may reach a point where a patching up and easing over of difficulties is all that can be done, but if any hope can be held out it can come only by the homœopathic and not the suppressive method of cure.

Throw out the disturbing elements if you can, and the blessed vital-force, so often maligned or denied to exist, will work out all that is possible of cure.

Right here is shown the reason why the simillimum bears frequent repetition so badly, for to start the vital-forces in the right direction is enough, and it is injurious to repress them while they are working, when their work flags, as shown by the cessation of improvement, it is often when some new obstacle is reached, calling for a new remedy; but just in proportion to the chronicity of the affection is the importance of watching the vital-forces and giving them only such stimuli as they can bear without repression and discouragement.

Hence the especial care necessary in avoiding careless or too frequent use of the deeper-acting, or "antipsoric" remedies, not because they themselves are more dangerous, but because they rouse the inmost springs of life, and work on a long chain of obstructions and distortions which must be straightened slowly and cautiously, if they are at all to be removed. If we decide that they cannot be removed, then it is wiser to turn our attention to the alleviation of less vital conditions, and so establish a sort of tolerance for the main incurable disease.

The homœopathician must use his tools with care in the presence of the eternal conflict between life and death.

